# Dissecting the Crosstalk between NRF2 Signaling and Metabolic Processes in Cancer

**DOI:** 10.3390/cancers12103023

**Published:** 2020-10-17

**Authors:** Janine M. DeBlasi, Gina M. DeNicola

**Affiliations:** 1 Department of Cancer Physiology, H. Lee Moffitt Cancer Center, Tampa, FL 33612, USA; janine.deblasi@moffitt.org; 2 Cancer Biology PhD Program, University of South Florida, Tampa, FL 33612, USA

**Keywords:** NRF2, cancer metabolism, KEAP1, NADPH, amino acids, lipids, oxidative stress

## Abstract

**Simple Summary:**

The stress-responsive transcription factor NRF2 (nuclear factor-erythroid 2 p45-related factor 2) directs cellular metabolic processes that can have diverse effects in the context of cancer. This review addresses how NRF2 and its negative regulator KEAP1 (Kelch-like ECH-associated protein 1) collectively modulate and respond to metabolism. We highlight NRF2-regulated processes relevant to the antioxidant system, cellular proliferation, and survival, including metabolism of amino acids, lipids, NADPH (reduced nicotinamide adenine dinucleotide phosphate), iron, and heme. We also review the stabilization of NRF2 by electrophiles, metabolites, and autophagy. Finally, we discuss topics that warrant further investigation into the KEAP1/NRF2 pathway’s role in tumor progression.

**Abstract:**

The transcription factor NRF2 (nuclear factor-erythroid 2 p45-related factor 2 or *NFE2L2*) plays a critical role in response to cellular stress. Following an oxidative insult, NRF2 orchestrates an antioxidant program, leading to increased glutathione levels and decreased reactive oxygen species (ROS). Mounting evidence now implicates the ability of NRF2 to modulate metabolic processes, particularly those at the interface between antioxidant processes and cellular proliferation. Notably, NRF2 regulates the pentose phosphate pathway, NADPH production, glutaminolysis, lipid and amino acid metabolism, many of which are hijacked by cancer cells to promote proliferation and survival. Moreover, deregulation of metabolic processes in both normal and cancer-based physiology can stabilize NRF2. We will discuss how perturbation of metabolic pathways, including the tricarboxylic acid (TCA) cycle, glycolysis, and autophagy can lead to NRF2 stabilization, and how NRF2-regulated metabolism helps cells deal with these metabolic stresses. Finally, we will discuss how the negative regulator of NRF2, Kelch-like ECH-associated protein 1 (KEAP1), may play a role in metabolism through NRF2 transcription-independent mechanisms. Collectively, this review will address the interplay between the NRF2/KEAP1 complex and metabolic processes.

## 1. Introduction

**Figure 1:**
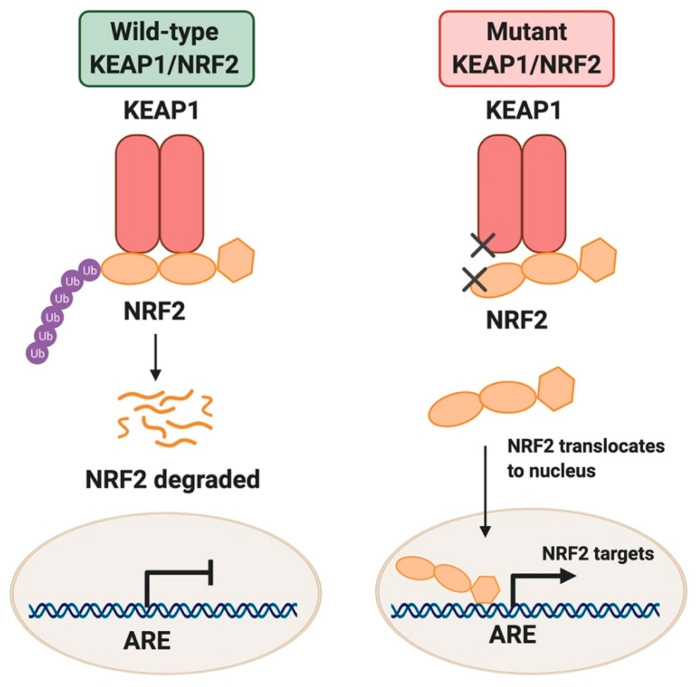
KEAP1/NRF2 regulation. Under non-stressed conditions KEAP1 directs ubiquitin-mediated degradation of NRF2, resulting in minimal transcription of NRF2 targets. Under oxidative or xenobiotic stress, or when the KEAP1/NRF2 pathway is mutated, NRF2 is stabilized and promotes transcription of antioxidant response element (ARE)-containing genes.

Over the past decade, advances in our understanding of the reprogramming of cellular metabolism in cancer cells have yielded insight into the complex biology of cancer. As this recently established cancer hallmark encompasses a plethora of pathways [1], we still lack a complete understanding of how metabolic pathways can be targeted in the treatment of cancer. The basic leucine zipper transcription factor NRF2 (gene name: *NFE2L2*) is emerging as a major regulator of cellular metabolism in both normal and cancer cells. NRF2 directs the transcription of both the antioxidant program and metabolic processes that support its function. NRF2 levels are controlled by its interaction with KEAP1, a substrate adaptor for a cullin 3 (CUL3)-based E3 ubiquitin ligase that targets NRF2 for ubiquitination and proteasomal degradation [2], as highlighted in Figure 1. While NRF2 protein levels are highly inducible in normal cells in response to oxidative and xenobiotic stress or electrophile exposure, constitutive NRF2 expression is observed in several cancers [3,4,5,6,7,8], including the squamous and adenocarcinoma subtypes of non-small cell lung cancer (NSCLC) [3], and squamous cancers of the esophagus, skin, larynx, and other tissues [6]. NRF2 stabilization most commonly occurs via activating mutations and copy number amplifications of the *NFE2L2* gene or inactivating mutations or deletions in *KEAP1* or *CUL3*. In cancer, somatic mutations in *NFE2L2* or its negative regulator *KEAP1* are associated with poor outcomes in patients [9] and often confer resistance to therapy through ROS and drug detoxification [10,11].

The roles of NRF2 in tumorigenesis are context-dependent [12,13,14,15,16,17,18,19,20,21,22], and likely influenced by different aspects of metabolism. The role of ROS metabolism is better defined, however, and has been well-characterized during cancer initiation, progression, and metastasis [23]. In healthy cells, activation of NRF2 promotes the transcription of anti-inflammatory and antioxidant genes that suppress the development of DNA damage and mutations that can initiate tumor formation [9]. In contrast, NRF2 supports the survival of transformed cells by protecting against oxidative damage to support their progression to more advanced-stage tumors. NRF2 loss can promote tumor cell migration and invasion through ROS-mediated epithelial-mesenchymal transition (EMT) [23,24]. NRF2 activation can also drive migration and invasion via the transcription factor BTB and CNC homology 1 (BACH1) in a heme metabolism-dependent manner [18]. These findings highlight the diverse roles that NRF2 plays during different stages of tumorigenesis in various contexts, and the distinct roles for ROS metabolism and metabolic regulation. Indeed, NRF2 regulates anabolic and catabolic metabolism, both of which can significantly impact tumorigenesis but are less well understood in the context of NRF2-driven cancer phenotypes. In this review, we will discuss NRF2-regulated metabolic processes and their role in cancer. Moreover, we will discuss the regulation of the KEAP1/NRF2 axis by metabolic perturbations and how this regulation also plays important roles in cancer.

## 2. Modulation of Metabolic Processes by NRF2

**Figure 2:**
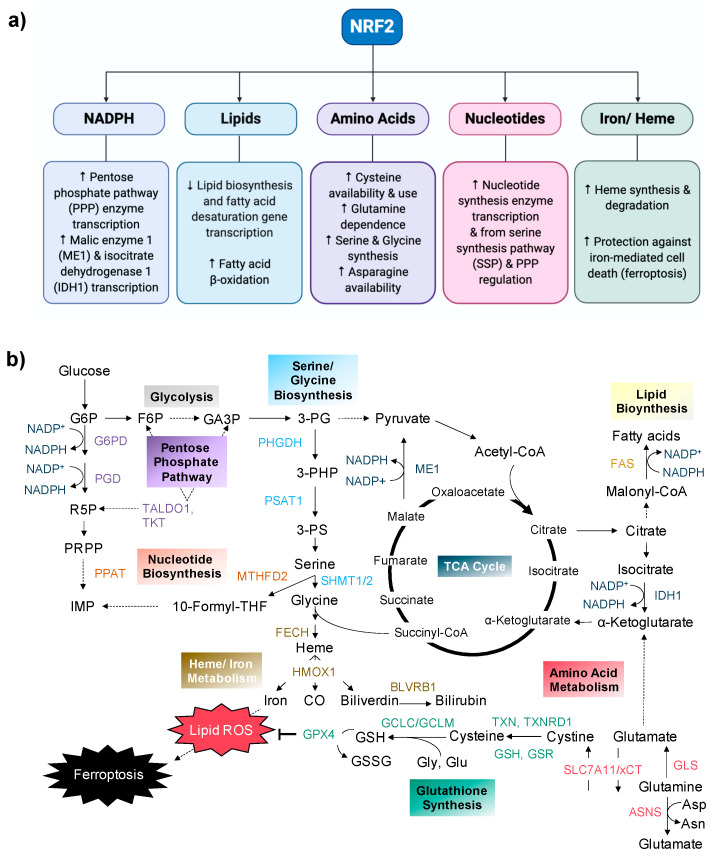
NRF2 directs numerous metabolic processes that impact cancer cell survival and proliferation, including NADPH production and the metabolism of lipids, amino acids, nucleotides, and iron/heme (**a**). These NRF2-regulated metabolic pathways are outlined in (**b**). NRF2 directs the transcription of numerous genes that encode for metabolic enzymes and transporters, including those involved in the pentose phosphate pathway (G6PD, PGD, TALDO1, TKT), nucleotide biosynthesis (PPAT, MTHFD2), serine/ glycine biosynthesis (PHGDH, PSAT1, SHMT1/2), heme/iron metabolism (FECH, HMOX1, BLVRB1), glutathione synthesis and utilization (TXN, TXNRD1, GSH, GSR, GCLC, GCLM, GPX4), amino acid metabolism (GLS, ASNS, SLC7A11/xCT), NADPH production (ME1, IDH1), and lipid biosynthesis (FAS). *Abbreviations*: Substrates: G6P, glucose-6-phosphate; R5P, ribose-5-phosphate; PRPP, phosphoribosyl pyrophosphate; IMP, inosine monophosphate; F6P, fructose-6-phosphate; GA3P, glyceraldehyde-3-phosphate; 3PG, 3-phosphoglycerate; 3-PHP, 3-phospho-hydroxypyruvate; 3-PS, 3-phosphoserine; 10-Formyl-THF, 10-formyltetrahydrofolate; CO, carbon monoxide; Asp, aspartate; Asn, asparagine; Glu, glutamate; Gly, glycine; ROS, reactive oxygen species; GSH, glutathione; GSSG, glutathione disulfide; TXN, thioredoxin; NADP^+^: nicotinamide adenine dinucleotide phosphate, oxidized; NADPH: nicotinamide adenine dinucleotide phosphate, reduced. Enzymes: G6PD, glucose-6-phosphate dehydrogenase; PGD, phosphogluconate dehydrogenase; TALDO1, transaldolase 1; TKT, transketolase; PPAT, phosphoribosyl pyrophosphate amidotransferase; MTHFD2, methylenetetrahydrofolate dehydrogenase 2; PHGDH, 3-phosphoglycerate dehydrogenase; PSAT1, phosphoserine aminotransferase 1; SHMT1/2, serine hydroxymethyltransferase; FECH, ferrochelatase; HMOX1, heme oxygenase 1; BLVRB1, biliverdin reductase B 1; ME1, malic enzyme 1; IDH1, isocitrate dehydrogenase1; FAS, fatty acid synthase; GLS, glutaminase; ASNS, asparagine synthetase; TXNRD1, thioredoxin reductase 1; GSR, glutathione reductase; GCLC, glutamate-cysteine-ligase catalytic subunit; GCLM, glutamate-cysteine ligase modifier subunit; GPX4, glutathione peroxidase 4. Other: SLC7A11/xCT: cystine glutamate antiporter.

In addition to orchestrating an antioxidant response to oxidative insults, there are emerging roles of NRF2 in promoting metabolic processes, including NADPH production [17,25,26,27], and the metabolism of lipids [27,28,29,30,31], amino acids (cysteine [19,32], glutamine [13,33], serine/glycine [12,34], asparagine [35]), nucleotides [25,36], and iron/heme [18,27,37,38,39], as outlined in Figure 2a,b. Although these processes play an important role in supporting the antioxidant response in healthy cells, they are hijacked by cancer cells to support proliferation and survival [40], warranting further investigation into the relationship between NRF2 and metabolism in tumorigenesis.

### 2.1. NADPH Production

The pentose phosphate pathway (PPP) is a major contributor to the cellular NADPH pool [41]. NADPH is critical for the synthesis of fatty acids, cholesterol, mevalonate pathway products [42], nucleotides, folate [41,43], and proline [40], all of which can support tumorigenesis. NADPH is also important for survival during ROS-mediated stress as a result of extracellular matrix (ECM) detachment [44]. Further, the PPP is often altered in cancers to support cell survival and proliferation [45]. The PPP comprises both irreversible and reversible reactions that are divided into an oxidative branch and a non-oxidative branch, respectively. The irreversible oxidative branch uses glucose 6-phosphate (G6P) to produce ribose-5-phosphate (R5P) and NADPH, the latter of which is critical for redox homeostasis. The reversible non-oxidative branch, in contrast, supports the production of only R5P for nucleotide synthesis, and serves to link glycolysis and the PPP [46]. The reversibility of this branch allows cells to synthesize R5P even when NADPH is high. NRF2 directly regulates the transcription of multiple PPP enzymes, including both oxidative PPP enzymes glucose-6-phosphate dehydrogenase (G6PD) and phosphogluconate dehydrogenase (PGD), and non-oxidative PPP enzymes transketolase (TKT) and transaldolase 1 (TALDO1) [25]. Additionally, NRF2 has been observed to indirectly regulate this pathway through the attenuation of microRNAs miR-1 and miR-206, thereby enhancing PPP gene expression [26]. Consequently, NRF2 can both directly and indirectly regulate the expression of PPP enzymes in both branches.

NRF2 also contributes to the NADPH pool via the transcriptional regulation of the NADPH-producing enzymes malic enzyme 1 (ME1) and isocitrate dehydrogenase 1 (IDH1) [25]. Given this apparent redundancy in NADPH-generating pathways, one might think that targeting any one individually may not dramatically affect the NADPH pool. Indeed, Zhao et al. reported that despite increased NRF2-mediated transcription of genes involved in NADPH regeneration, the oxidative PPP actually contributed less to the NADPH pool, and consequently was less essential for the growth of KEAP1 mutant cells [47]. Overall, these studies demonstrate the importance of KEAP1 mutational status when evaluating sensitivity to PPP inhibition in a therapeutic context. However, recent work suggests that the oxidative PPP plays a unique role in folate metabolism [41]. Chen et al. demonstrated that while the PPP, IDH1 and ME1 could all support cellular proliferation, only the PPP could maintain a normal NADPH/NADP+ ratio. Deletion of G6PD resulted in high NADP^+^, leading to dihydrofolate reductase (DHFR) inhibition, and ultimately, impairment of folate-mediated biosynthesis in colon cancer cells, unveiling an important connection between NADPH and folate metabolism [41]. Supportingly, Mitsuishi et al. found that silencing the PPP enzymes G6PD or TKT reduced tumor growth in a KEAP1 mutant NSCLC xenograft model in a similar manner to silencing NRF2 [25]. Moreover, Best et al. observed that Keap1 mutant mouse lung tumors expressed high levels of Taldo1 and were more sensitive to inhibition of the PPP enzyme Pgd with 6-AN compared with their wild-type counterparts [17]. Collectively, these studies highlight the regulation of the PPP by NRF2, which represents a critical metabolic vulnerability in vivo.

### 2.2. Lipid Metabolism

Lipid synthesis is a highly NADPH-consuming process that competes with cellular antioxidant systems for NADPH. NRF2 activation suppresses fatty acid synthesis and desaturation [27,29] to increase NADPH for detoxification and anabolism in murine liver. While hepatocyte-specific deletion of Keap1 suppressed the expression of fatty acid synthesis and desaturation enzymes, deletion of Nrf2 increased their expression [27]. A reduction in fatty acid synthesis enzymes and triglyceride accumulation was also observed in the livers from a Keap1 hypomorphic mouse model, which has elevated Nrf2 expression, fed a high-fat diet [29]. Although the ability of NRF2 to suppress lipogenesis has been established in liver, it is unclear whether this also occurs in tumor cells or how this would be compatible with the growth of a tumor with constitutive NRF2 stabilization. The co-occurrence of other mutations in tumor suppressors and oncogenes, however, may alleviate this potential block. For example, in NSCLC, KEAP1 mutant tumors tend to be highly enriched for alterations in serine/threonine kinase 11 (STK11), a gene that encodes for the liver kinase B1 (LKB1) [48]. It has been suggested that KEAP1 and STK11 mutations may cooperate; Stk11 deletion in mouse embryonic fibroblasts (MEFs) causes increased glucose-dependent lipid biosynthesis and overall lipid content [49]. This work suggests that LKB1-deficient tumor cells can divert NADPH into anabolic processes, potentially compensating for the lipid synthesis block conferred by NRF2. Indeed, Jeon and colleagues demonstrated that LKB1-deficient cancer cells increased NADPH consumption by fatty acid synthase (FAS) due to loss of AMPK-mediated regulation [50]. Furthermore, NRF2 has been recently reported to enhance mitochondrial fatty acid oxidation (FAO) [28]. When Nrf2 is constitutively active as a result of Keap1 knockout, mitochondrial oxidation of both short-chain and long-chain fatty acids was increased; in contrast, Nfe2l2 knockout decreased their oxidation [28]. It was also reported that expression of an activating Nfe2l2 mutant (Nrf2^E79Q^) decreased adipogenesis in keratin 14 (KRT14)-positive mouse tissue [30]. Further, RNA sequencing (RNAseq) profiling demonstrated that Nrf2^E79Q^ esophageal epithelial cells upregulated peroxisome proliferator activated receptor delta (PPARδ), which regulates FAO [30]. More recently, Suzuki and colleagues showed the contribution of Nrf2 to weight gain in mice during space travel, where systemic Nfe2l2 knockout decreased FAO-related gene expression and white adipose tissue homeostasis, in agreement with previous studies demonstrating the importance of NRF2 in lipid metabolism [31]. Overall, the role of NRF2-regulated fatty acid metabolism in tumorigenesis requires further investigation, especially in the context of co-occurring mutations.

### 2.3. Amino Acid Metabolism

#### 2.3.1. Cysteine, Cystine and Glutathione

Cysteine is a semi-essential amino acid that can be taken up by cells in both its reduced (cysteine) and oxidized (cystine) forms. Cysteine transport is poorly characterized in both normal and tumor tissues, and may involve the alanine-serine-cysteine (ASC), L-amino acid, and/or X_AG_ family of transporters [51,52,53]. Alternatively, cysteine can be made by de novo synthesis through the reverse transulfuration pathway in some tissues [54]. Further, cystine is transported via system x_c_-, the cystine/glutamate antiporter [55]. xCT, the protein encoded by SLC7A11, is a subunit of system x_c_-, and is upregulated in many cancer types, including NSCLC, triple-negative breast cancer, and glioblastomas [56,57,58,59,60,61]. NRF2 increases intracellular cysteine availability through multiple mechanisms. NRF2 promotes the transcription of xCT to facilitate cystine entry into the cell [32]. NRF2 also transcriptionally regulates thioredoxin (TXN) and thioredoxin reductase 1 (TXNRD1), which contribute to the reduction of cystine to cysteine [62,63].

NRF2 promotes the use of cysteine for the synthesis of the antioxidant glutathione (GSH) for ROS detoxification. NRF2 increases the activity of the enzyme that catalyzes the first step of GSH synthesis, gamma-glutamyl-cysteine-ligase (GCL), a heterodimeric enzyme comprised of GCL catalytic subunit (GCLC) and GCL modifier subunit (GCLM) [64,65,66,67]. GCL facilitates the conjugation of cysteine and glutamate to produce γ-glutamylcysteine, a precursor for GSH [68]. Although the regulation of cysteine metabolism by NRF2 robustly increases GSH levels following NRF2 activation, it is less clear whether NRF2-mediated cysteine accumulation promotes the synthesis of other cysteine-derived metabolites.

We recently reported that cysteine accumulation mediated by NRF2 was a metabolic vulnerability in NSCLC cells as a consequence of stabilization of cysteine dioxygenase 1 (CDO1) and increased entry of cysteine into the taurine synthesis pathway [19]. This led to wasteful and toxic product formation and depletion of NADPH as a consequence of excessive cystine reduction, which impaired NSCLC proliferation and antioxidant function. Notably, CDO1 is epigenetically silenced in NSCLC, particularly in KEAP1 mutant adenocarcinomas. Consequently, not all NRF2-regulated processes are favorable.

#### 2.3.2. Glutamine

NADPH is not the only cost of cystine import. Because xCT is a cystine-glutamate antiporter, cystine import must be matched by an equimolar amount of glutamate export. Accordingly, NRF2 active cells are in a glutamate-deficient state as a consequence of elevated xCT activity, which limits TCA cycle anaplerosis and increases reliance on glutamine catabolism to glutamate to support xCT flux [13,33]. In agreement with this metabolic vulnerability, KEAP1 mutant NSCLC cell lines, PDX models and Keap1-deficient mouse tumors demonstrate increased sensitivity to glutaminase inhibition with CB-389 [13,57]. Glutaminase inhibition has also been reported to prevent growth of recurrent breast tumor cells in a NRF2-dependent manner [36]. In support of this NRF2-mediated dependence on glutamine, it has also been reported that cancer cells with high antioxidant capacity are dependent on non-essential amino acids (NEAAs) driven by xCT-mediated excretion of glutamate required for NEAA synthesis [69]. This study also observed therapeutic efficacy in a Keap1 mutant mouse model treated with an inhibitor of glutaminase or asparaginase.

The connection between the KEAP1/NRF2 pathway and glutamine metabolism is not surprising, given that NRF2 upregulates glutaminase, which generates glutamate from glutamine [70]. Recent work has also shown that KEAP1 mutant lung cancer cells are dependent on glutamine and the glutamine transporter ASCT2 (encoded by SLC1A5) [13]. It has also been suggested that generation of TCA cycle intermediates by alternative pathways, such as IDH1 and ME1, may contribute to NRF2-mediated glutamine dependence [71]. Furthermore, siRNA depletion of NRF2 in KEAP1 mutant NSCLC cells decreases incorporation of glutamate into GSH [25], suggesting that glutamate production from glutamine could support GSH synthesis. These studies highlight a metabolic vulnerability in NRF2-hyperactive cancer cells.

#### 2.3.3. Serine/Glycine

Serine and glycine are NEAAs that contribute to diverse macromolecules within normal and cancer cells, including cysteine, sphingolipids, phospholipids, and nucleotides, among others [72,73]. Serine and glycine can be obtained from extracellular sources or synthesized de novo from glucose via the serine synthesis pathway (SSP). The SSP catalyzes the metabolism of the glycolytic intermediate 3-phosphoglycerate (3PG) by phosphoglycerate dehydrogenase (PHGDH) in the first and rate-limiting step. SSP-derived serine is subsequently metabolized to glycine in a reaction mediated by serine hydroxymethyl transferase (SHMT). NRF2 plays an important role in the regulation of SSP enzymes including PHGDH, phosphoserine aminotransferase 1 (PSAT1), and SHMT2 via the amino acid starvation-responsive activating transcription factor 4 (ATF4) [12]. Suppression of serine synthesis in KEAP1 mutant NSCLC cell lines impaired the synthesis of GSH and nucleotides, and depleted cellular NADPH levels [12]. KRAS/KEAP1 mutant NSCLC cell lines were also found to depend on GLUT8 (SLC2A8) for serine biosynthesis, suggesting that serine synthesis addiction can also be targeted at the level of glucose availability [74]. Beyond lung cancer, NRF2 SUMOylation in hepatocellular carcinoma (HCC) was shown to promote de novo serine synthesis via PHGDH upregulation, leading to serine accumulation and contributing to HCC maintenance [34]. Finally, work has also linked serine availability and PHGDH to sphingolipid metabolism, with very low serine levels promoting toxic deoxysphingolipid synthesis [75,76,77]. In alignment with these studies, earlier work demonstrates the importance of serine in supporting mitochondrial function through ceramide metabolism [78]. Additional work is needed to determine whether sphingolipid metabolism is influenced by NRF2 activation in cancer.

Beyond NRF2 regulation, SSP activity is increased in diverse cancer types as a consequence of PHGDH amplification, overexpression, and posttranscriptional regulation [12,79,80,81,82,83,84,85]. To potentially exploit this, PHGDH inhibitors are being explored as treatments for cancer. PHGDH inhibition has demonstrated preclinical utility in xenograft models of colon cancer [77], as well as xenografts of breast cancer and renal cell carcinoma exhibiting brain metastases [86]. The latter findings are also important for NSCLC patients, who can develop brain metastases [87,88,89], and thus may respond to PHGDH inhibition. Both PHGDH inhibitor treatment and whole-body Phgdh knockdown are non-toxic in mice, which suggests that PHGDH inhibitors may be safe for patients as long as adequate serine and glycine are present in the diet [75]. In summary, these studies demonstrate inhibition of the SSP as a potential therapeutic modality in cancer.

#### 2.3.4. Asparagine

Asparagine is another NEAA that can be obtained from extracellular sources or synthesized de novo. Asparagine synthetase (ASNS) catalyzes the transamidation of aspartate and glutamine to produce asparagine [90]. ASNS, which like SSP enzymes is regulated by ATF4, plays an important role in maintaining asparagine levels under nutrient limiting conditions [91]. Further, asparagine has been reported to protect against cell death from glutamine depletion, implicating its role as a metabolite used by cells to coordinate response to metabolic stress [92]. NRF2 plays an important role in controlling asparagine availability through the regulation of ASNS. NRF2 regulated the binding of ATF4 to the ASNS promoter in KEAP1 mutant cells [12]. Furthermore, NRF2 was essential for KRAS-mediated regulation of ATF4 under nutrient stress. In addition, deletion of KEAP1 enhanced the induction of ATF4 and ASNS following glutamine deprivation [35]. Because asparagine depletion by L-asparaginase is a potential therapeutic strategy for the treatment of tumors, these studies suggest that the regulation of ASNS by NRF2 to increase asparagine availability may influence tumor response to L-asparaginase.

### 2.4. Nucleotide Metabolism

NRF2 supports nucleotide synthesis through both metabolic mechanisms and the transcriptional regulation of nucleotide synthesis enzymes. NRF2 regulates the activity of the PPP, which supports nucleotide metabolism by producing the R5P sugar backbone for nucleotide synthesis, and NADPH for the reduction of ribonucleotides to deoxyribonucleotides. Additionally, the SSP provides glycine for purine synthesis and one carbon units for the synthesis of both purines and pyrimidines. Furthermore, NRF2 directly regulates the expression of enzymes involved in de novo nucleotide synthesis. NRF2 knockdown decreases the mRNA expression of phosphoribosyl pyrophosphate amidotransferase (PPAT) and methylenetetrahydrofolate dehydrogenase 2 (MTHFD2) [25]. PPAT adds an amine group from glutamine to phosphoribosyl pyrophosphate (PRPP) in the first step of purine synthesis, while MTHFD2 plays a critical role in the synthesis of the 10-formyl-tetrahydrofolate molecules that are added to the purine ring in downstream steps. While nucleotide synthesis can support cancer cell proliferation, recent work has also shown that NRF2 signaling promotes transcriptional and metabolic reprogramming to support redox homeostasis and increase de novo nucleotide synthesis during breast tumor recurrence [36]. Using in vivo CRISPR screening, Pgd of the oxidative PPP and Ppat were shown to be downstream NRF2 targets required for recurrent tumor growth. These studies highlight the importance of NRF2 in directing nucleotide metabolism for both tumor growth and recurrence.

### 2.5. Iron/Heme

Iron is essential for cellular metabolic processes, but reactive iron can promote the formation of ROS, leading to cellular damage and death. To limit the detrimental effects of free iron, NRF2 regulates many genes involved in iron and heme metabolism, including heme oxygenase 1 (HMOX1) and the genes encoding the iron storage protein ferritin, ferritin heavy chain (FTH1) and ferritin light chain (FTL) [39]. Importantly, many interactions between iron and oxygen occur via a porphyrin-bound form of iron, heme, which is in the center of several metabolic enzymes [93]. NRF2 promotes the synthesis of heme through the regulation of ferrochelatase (FECH), and its transport via SLC48A1 [37]. Surprisingly, NRF2 also promotes the degradation of heme through HMOX1 and biliverdin reductase B (BLVRB) [27,38]. HMOX1 encodes for a cytoprotective enzyme that catalyzes heme degradation, resulting in production of iron, biliverdin, and carbon monoxide [94]. BLVRB1 subsequently reduces biliverdin to bilirubin, which is excreted. Many cellular processes rely on iron and heme, and the role of these enzymes in NRF2-mediated tumor initiation and progression is poorly understood.

In cancer, NRF2-mediated disruption of HMOX-1 and ferritin signaling can impact cancer cell proliferation, angiogenesis, metastasis, and response to therapy, which is usually influenced by the amount of ROS and iron present [95]. HMOX-1 and NRF2 induction can protect against ferroptosis, an iron-mediated form of cell death. In HCC, Sun and colleagues reported that NRF2 protected HCC cells against ferroptosis via p62-mediated NRF2 stabilization. In this study, the anti-ferroptotic activity of NRF2 was dependent on NQO1, HMOX1, and FTH1 [96]. NRF2 also transcriptionally regulates glutathione peroxidase 4 (GPX4) [97], a key regulatory factor for ferroptosis that mitigates lipid peroxidation and consequent ferroptosis [98]. Another key mediator of iron/heme metabolism is the transcription factor BACH1. Heme binds to BACH1, resulting in nuclear export and subsequent ubiquitin-mediated degradation, thereby limiting the amount of BACH1 available to bind DNA in complex with NRF2 [99]; in contrast, when heme levels are low, BACH1 antagonizes the ability of NRF2 to activate HMOX1 to promote heme degradation [100]. However, BACH1 plays important roles in cancer beyond its role in heme homeostasis. In a mouse model of lung adenocarcinoma, Keap1 deletion/Nrf2 activation promoted metastasis via Hmox1, which promoted the degradation of heme to induce the accumulation of Bach1, which regulated a battery of pro-metastatic genes [18]. Collectively, these studies highlight the ways in which NRF2 can modulate iron/heme metabolism to impact tumor phenotypes.

## 3. Metabolic Pathways that Stabilize NRF2

**Figure 3:**
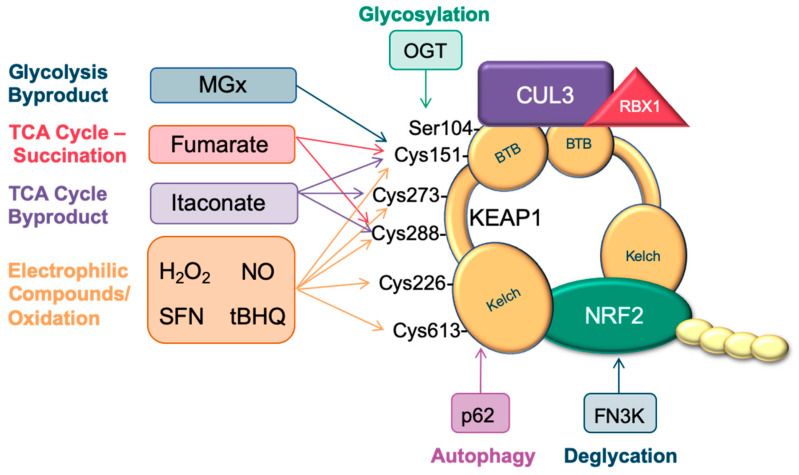
NRF2 is activated by oxidants, signaling molecules, and metabolites. **KEAP1:** KEAP1 is glycosylated by O-GlcNAc transferase (OGT) at serine 104. The glycolysis byproduct methylglyoxal (MGx) mediates a crosslink between cysteine 151 with arginine 135, activating the NRF2 transcriptional program. Further, NRF2 activation can result from fumarate-mediated succination of cysteines 151 and 288. The TCA cycle byproduct itaconate can also activate NRF2 by reacting with cysteines 151, 273, and 288. Hydrogen peroxide (H_2_O_2_), nitric oxide (NO), sulforaphane (SFN), and *tert*-butyl hydroquinone (tBHQ) can activate NRF2 by modifying cysteine residues 151, 273, 288, 226, and 613. The autophagy adaptor sequestome 1, p62, activates NRF2 by binding to the Kelch domain region of KEAP1. **NRF2:** NRF2 is destabilized by glycation, which is reversed by the enzyme Fructosamine-3-kinase (FN3K).

While NRF2 can direct many important pathways in metabolism, it is also regulated by various metabolic processes, including oxidative stress/electrophiles, fumarate, glucose, itaconate, and autophagy. Figure 3 highlights the various metabolites and other electrophiles that activate NRF2 by modifying amino acid residues on KEAP1 or NRF2.

### 3.1. Electrophiles

The classic mechanism of NRF2 activation is in response to electrophilic insults in the cell [39]. Under non-stressed conditions, KEAP1-mediated NRF2 degradation limits the amount of NRF2 available to translocate to the nucleus to induce target gene expression [2]. Under stressed conditions (i.e., high ROS levels), KEAP1-mediated degradation of NRF2 is inhibited, and NRF2 accumulates in the nucleus where it can promote transcription of ARE-containing genes involved in oxidative damage prevention and redox homeostasis [38]. Among NRF2 target genes are those encoding for enzymes involved in phase I/II/III detoxification, GSH synthesis, NADPH production, survival, and iron sequestration [39]. NRF2 is usually activated by metabolic stressors that react with cysteine residues on KEAP1, of which the most well-characterized sensor residue is C151 [101]. C151 is located in KEAP1’s bric à brac (BTB) domain and is highly reactive to known NRF2 inducers, including nitric oxide (NO), sulforaphane (SFN), *tert*-butyl hydroquinone (tBHQ), and hydrogen peroxide (H_2_O_2_) [101,102]. Further, it was determined that C273 and C288 in KEAP1’s linker domain are required for KEAP1-mediated repression of NRF2 [101]. Importantly, many diseases cause oxidative stress that can result in modification of KEAP1 cysteine residues and prompt NRF2 activation, including cancer, diabetes, neurogenerative disease, and inflammation-related diseases, among others [2]. Overall, the KEAP1-NRF2 axis facilitates redox homeostasis and cytoprotection.

### 3.2. Fumarate

Fumarate hydratase (FH) is a TCA cycle enzyme that catalyzes the hydration of fumarate to malate and is considered a tumor suppressor; the inactivation of FH is associated with leiomyomata, renal cysts, and tumors [103]. Although fumarate is structurally similar to succinate and 2-hydroxyglutarate, and consequently can function as an allosteric regulator of oxoglutarate-dependent oxygenases such as HIF prolyl hydroxylases [104], fumarate is also an electrophile. Indeed, fumarate modifies cysteine residues in KEAP1, impairing its ability to degrade NRF2 [103]. Adam et al. found that the NRF2 activation from FH deficiency was caused by succination of KEAP1’s redox-sensitive cysteine residues, rather than global oxidative stress. Thus, NRF2 activation is predicted to occur early in renal tumorigenesis during hyperplastic cyst development as a result of FH inactivation [103]. Interestingly, FH deficiency is synthetically lethal with inhibition of the NRF2 target gene *HMOX1*, posing a potential therapeutic option for patients with renal tumors [105]. The heme degradation pathway allows FH-deficient cells to funnel excess TCA cycle intermediates into secretable, non-toxic bilirubin, thereby promoting cell viability. Interestingly, another NRF2-regulated enzyme in heme degradation, BLVRB, was also predicted to be synthetically lethal with FH loss in this study. Collectively, these findings suggest that the KEAP1/NRF2 pathway may function to sense TCA cycle defects and respond to restore metabolic homeostasis.

### 3.3. Glucose

The reactive metabolite, methylglyoxal (MGx), is produced as a byproduct of glycolytic metabolism. Recently, Bollong et al. demonstrated that inhibition of the glycolytic enzyme phosphoglycerate kinase 1 (PGK1) resulted in MGx accumulation and activation of the NRF2 transcriptional program as a consequence of a MGx-mediated methylimidazole crosslink between cysteine and arginine residues in KEAP1 [106]. This finding is very reminiscent of the regulation of KEAP1 by fumarate, as NRF2 plays a critical role in the cellular protection against the toxic effects of MGx. MGx detoxification by the glyoxylase pathway requires GSH [107], an important consequence of NRF2 activation. Furthermore, earlier work demonstrated that NRF2-mediated protection against MGx was primarily through promotion of GSH synthesis in the context of neuronal MGx-induced carbonyl stress [108]. Finally, Mitsuishi and colleagues’ seminal work suggests that NRF2 can contribute to directing glucose away from metabolites like MGx and into the PPP as described earlier [25]. Overall, these studies illustrate how the KEAP-NRF2 pathway responds to endogenous metabolic stress and holds translational potential for diseases linked to these pathways.

In addition to being modified by MGx, KEAP1 can also be glycosylated, establishing an important link between NRF2 signaling and nutrient-sensitive modification [109]. Chen and colleagues identified KEAP1 as a substrate of O-GlcNAc transferase (OGT), which adds the post-translational modification (PTM) O-GlcNAcylation at S104. This finding is not only important in the context of the KEAP1-NRF2 pathway, but also extends to proteostasis and nutrient sensing. The S104 residue on KEAP1 is conserved among other Kelch-like (KLHL) family proteins, which suggests that this modification may be relevant for the response of other proteins to redox stress. Further, O-GlcNAcylation requires a nucleotide-sugar (UDP-GlcNAc) from the hexosamine biosynthetic pathway, thus linking KEAP1 modification to cellular nutrient status, and supporting the established role of OGT in nutrient sensing [109]. Furthermore, it was recently shown that NRF2 is heavily glycated, which is a consequence of the non-enzymatic reaction of reducing monosaccharides like glucose to basic amino acids. Glycation is reversible, which is mediated by Fructosamine-3-kinase (FN3K). Sanghvi et al. found that glycation of NRF2 decreased both protein stability and small musculoaponeurotic fibrosarcoma (MAF) protein binding, thereby reducing NRF2 transcriptional output [110]. This study suggests that NRF2 responds to sugar availability and that targeting FN3K may be a potential therapeutic strategy for NRF2 active cancers. In summary, these studies are important for showing ways in which NRF2 is stabilized in response to glucose-related modifications, and how they might be targeted therapeutically.

### 3.4. Itaconate and the Immune System

NRF2 activation not only promotes the proliferation and survival of cancer cells, but it is also critical for proper immune function. NRF2 suppresses macrophage inflammatory response by blocking the transcription of proinflammatory cytokines, including IL-6 and IL-1β; this was found to be independent of ROS and the canonical NRF2-binding motif in cytokine genes [111]. The regulation of the immune microenvironment also extends beyond macrophages to other myeloid populations and regulatory T cells [112,113], which can influence tumor progression and metastasis. Importantly, NRF2 activation within the tumor microenvironment suppressed the progression of mutant *Kras*-driven lung tumors [114]. It is not surprising that immune populations can direct their own function through NRF2 regulation. The metabolite itaconate is made from decarboxylation of the TCA cycle intermediate cis-aconitate [115] and promotes NRF2 stabilization in macrophages via itaconate-mediated alkylation of cysteine residues on KEAP1, which allows for NRF2 activation of antioxidant and anti-inflammatory target genes [116]. In turn, NRF2 stabilization enables the ability of itaconate to abrogate inflammation and regulate type I interferons (IFN) [116]. The role of itaconate/NRF2 is complex and may be context-dependent, however. Recent work has shown that itaconate-mediated NRF2 activation in hepatocytes can protect against hepatic ischemia-reperfusion injury [117], illustrating a non-immune cell mechanism of protection. Additional work also suggests that itaconate and the anti-inflammatory effects of NRF2 may be stimulus-dependent in certain contexts, and may depend on whether itaconate is endogenous or exogenous [118]. Sun and colleagues observed that endogenous itaconate was not required for a NRF2 response to particulate matter (PM), which comprises air pollution, and that only exogenously applied itaconate could induce NRF2 activation. Further, they observed that NRF2 was actually dispensable for the anti-inflammatory effects of exogenous itaconate, suggesting that NRF2 does not significantly contribute to regulation of the anti-inflammatory effect of itaconate in response to PM. Further investigation into the complex relationships between NRF2, itaconate, and inflammation is warranted to determine in which contexts they may be therapeutically exploited.

### 3.5. Autophagy

The role of autophagy in cancer is somewhat paradoxical; in some cases, autophagy can help to remove cancer cells, whereas in other cases tumor cells can harness autophagy as a protective process during nutrient deprivation, oxidative stress, and other stress-inducing factors [119,120,121]. The autophagy adaptor sequestome 1 (p62) can promote NRF2 activation via competitive binding to KEAP1 to increase the free NRF2 pool [122] and also by promoting autophagic degradation of KEAP1 [123]. Because p62 is also a transcriptional target of NRF2, NRF2 can increase its own activity in a positive feedback loop through increased p62 transcription. Autophagy deficiency can be tumor promoting through NRF2 activation. In mice, liver-specific deletion of the essential autophagy gene *Atg7* promoted accumulation of p62, Nrf2 activation, and the development of HCC [124]. Moreover, ectopic p62 expression was sufficient to promote NRF2 activation and HCC development [125], demonstrating a causal role for p62 accumulation in HCC development downstream of autophagy deficiency. Inflammation-induced autophagy impairment in PDAC also promoted p62-mediated activation of NRF2, which promoted neoplasm progression via NRF2-mediated MDM2 induction [126]. While NRF2 activation by p62 in cancers is protumorigenic, NRF2 also plays a critical role in the cellular response to autophagy deficiency in normal tissues. NRF2 protected against small intestine damage and animal death following whole body *Atg7* and *Trp53* deletion [127], suggesting that NRF2 activation by p62 is important for cellular adaptation and homeostatic control. NRF2 can regulate the expression of certain proteasome subunits, which may explain how NRF2 is protective in the context of autophagy impairment [128]. Furthermore, NRF2 is also stabilized in response to proteasome impairment, and can subsequently respond to stress in concert with autophagy [129]. More recent work also suggests that NRF2 may contribute to aggrephagy - a form of protein degradation that directs misfolded proteins to the microtubule-organizing center for autophagic degradation during proteasomal stress. In cells with low proteasome activity, NRF2 is stabilized and can facilitate aggresome formation, mitigating this stress [130]. Collectively, these studies highlight an important role for NRF2 in responding to autophagic impairment and/or proteasomal stress. 

## 4. Future Perspectives

Much focus has been on the role of NRF2 in mediating the metabolic outputs of KEAP1 modification, but KEAP1 has other substrates and interacting partners, some with the potential to regulate metabolism. Very few true KEAP1 substrates have been identified and these include the DNA-repair protein partner and localizer of BRCA2 (PALB2) [131] and the DNA helicase subunit mini-chromosome maintenance complex component 3 (MCM3) [132]. Many more interacting partners have been found, including phosphoglycerate mutase 5 (PGAM5), which forms a complex containing both NRF2 and KEAP1, where KEAP1 binds to both proteins through their E(S/T)GE motifs [133]. PGAM5 is a serine/threonine and histidine [134] phosphatase that localizes on the mitochondrial outer membrane and can affect mitophagy, necroptosis, and overall mitochondrial dynamics [133,135]. Although PGAM5 is not a NRF2 target, NRF2 is still required for this complex, which serves to protect mitochondrial motility through suppression of dominant-negative KEAP1 activity [136]. Recently, PGAM5 was implicated in a novel ROS-sensitive cell death pathway termed “oxeiptosis” [137]. Further, it was reported that PGAM5’s dephosphorylation of ME1 increases NADPH production, lipid synthesis, and colorectal tumorigenesis [138]. Overall, PGAM5 is important for metabolism and may have cellular implications upon mutations in *KEAP1*. Further investigation into metabolic roles of KEAP1 independent of NRF2 transcription is warranted.

## 5. Conclusions

While it has long been known that NRF2 is imperative to cellular homeostasis via its role in the antioxidant response, the regulation of metabolic pathways at the interface between antioxidant defense and cellular proliferation is an emerging and essential function of NRF2. The recent identification of the regulation of NRF2 by metabolic perturbations suggest that NRF2 plays a key role in metabolic homeostasis beyond supporting the antioxidant response. While some of these perturbations have been linked to cancer (e.g., fumarate hydratase mutations, p62 accumulation), further work is needed to determine whether others also play a causal role in tumorigenesis. Moreover, more work is needed to characterize the necessity for NRF2-regulated metabolic pathways for tumor initiation and progression. Much of the focus thus far has been on the antioxidant response and metabolic pathways directly linked to ROS metabolism (e.g., the pentose phosphate pathway). Furthermore, a deeper understanding of the metabolic vulnerabilities, such as glutaminase inhibitor sensitivity, and metabolic liabilities, such as CDO1 accumulation, associated with NRF2 activation will provide new opportunities for therapy. It is becoming clear that not all NRF2-regulated metabolic processes are favorable and can be exploited to impair tumor growth and viability. However, care must be taken to consider the role of NRF2 and its downstream processes in the microenvironment, where it influences immune function and plays other roles. Finally, the metabolic outputs of KEAP1 modification that are NRF2-independent are completely unexplored and have the potential to contribute to both normal and tumor metabolism. A more comprehensive understanding of these factors will be critical for understanding and treating *KEAP1/NFE2L2* mutant tumors.
